# Research on Environmental Regulation and Green Total Factor Productivity in Yangtze River Delta: From the Perspective of Financial Development

**DOI:** 10.3390/ijerph182312453

**Published:** 2021-11-26

**Authors:** Jia Li, Decai Tang, Acheampong Paul Tenkorang, Zhuoran Shi

**Affiliations:** 1 College of Economics and Management, Nanjing University of Aeronautics and Astronautics, Nanjing 211106, China; 2 School of Law and Business, Sanjiang University, Nanjing 210012, China; 3 School of Management Science and Engineering, Nanjing University of Information Science & Technology, Nanjing 210044, China; 20215124001@nuist.edu.cn; 4 Reading Academy, Nanjing University of Information Science & Technology, Nanjing 210044, China; szr1330201602@163.com

**Keywords:** environmental regulation, green total factor productivity, financial development, influence effect

## Abstract

This paper employs the global Malmquist Luenberger (GML) index and the System Generalized method of moments (GMM) estimation method to investigate the influence of both environmental regulation and financial development on green total factor productivity in 41 cities of the Yangtze River Delta (YRD) in China from 2003–2019. We select the relevant input-output data to measure the green total factor productivity (GTFP) and its decomposition index including undesirable output. The results show that the GTFP and its decomposition index in the YRD have a slow fluctuating upward trend. The YRD mainly depends on improving the level of technological progress and environmental governance to promote the improvement of regional economic green development level. The empirical research results show that there is an inverted U relationship between environmental regulation and GTFP in the YRD, too strict environmental regulation will inhibit the growth of green total factor productivity. By adding control variables, the inflection point of environmental regulation is 0.5034, which is lower than that without control variables. There is a strong interaction and superposition effect between financial development and environmental regulation, which is closely related to the established financial cooperation mechanism, perfect financial system arrangement and cross-regional financial cooperation platform in the YRD. Government intervention should be reduced, the introduction of foreign capital should be controlled appropriately, foreign capital should be guided to green industries, and the use efficiency of foreign capital should be improved. This paper holds that we should pay attention to the strength of environmental regulation, prevent overcorrection, increase the guidance of credit funds, deepen the reform of the financial system, appropriately intervene in the market by the government, strengthen the guidance of foreign capital, and promote the development and transformation of the green economy in the YRD region with the help of several policies.

## 1. Introduction

Green sustainable development is the trend of human development and has become a consensus all over the world. Its essence is the coordinated development of ecology and economy as the core. Green economy/green growth is a new terminology for what is known after 40 years as ecological modernization [1]. For policy makers, the development of a green economy presents opportunities and challenges not only for the central government, but also for provincial and city governments [2]. Establishing and improving the level of green development economic system and promoting the comprehensive green transformation of economic and social development are the basic policies to solve the ecological problems of resources and environment. In this process, it is important to provide finance while enhancing the resource efficiency and reduce the impact on the environment and the global climate [3]. Consequently, balanced adherence to financial, economic, and environmental criteria is the main foundation.

Does environmental regulation increase or decrease the development of Green Economy by encouraging the development of finance? In this vein, it is important to understand the measurement of green economy as well as the determinants of green economic development. In comparison with the considerable attention devoted to understanding the effect of financial development and environmental regulation on green economy respectively, much less attention has been devoted to understanding the nonlinear influence of environmental regulation on the development of green economy [4,5], and even less is known about whether the financial development and environmental regulation can play superposition effect on the development of green economy.

We need to accelerate the transformation and upgrading of traditional industrial structure and eliminate highly polluting enterprises. We also need to constantly improve environmental protection standards, strengthen environmental regulation, and force the optimization and upgrading of economic structure to promote the green transformation of economic development. Environmental policies are affected by technological and financial developments, financial development promotes energy and environmental-technological growth [6]. With the low efficiency of financial development, the environmental regulation is not conducive to technological innovation, while environmental regulation can effectively promote technological innovation with a high efficiency of financial development. There are dissimilarities in the progress across countries/regions on the front of economic growth in synchronization with the sustainable development. To achieve global sustainable development, it is critical for countries/regions that are underperforming on this front to observe and learn from those that are outperforming [7]. Researchers have long endeavored to evaluate green economy development efficiency as well as the influencing factors. It is very necessary to study the relationship between green economy development and influencing factors, which will help to formulate an inclusive, consistent and efficient action plan.

In recent years, China’s economy has maintained rapid growth, but there are still some problems affecting the improvement of the quality of economic growth. A typical representation of such a problem is the existence of an unbalanced and uncoordinated regional development. The extensive growth model of high investment, high consumption, high pollution, low quality, low efficiency, and low output has caused serious ecological and environmental pressure. With this series of associated problems, green and low-carbon development has become an inevitable choice. Realizing green and sustainable development with low energy consumption, low pollution and high efficiency has become an important path for China’s economic development at present and in the future. Facing the multiple constraints of resources, environment, the driving force of sustainable development and other factors, how to improve China’s total factor productivity through green development and transformation is not only a practical problem to be solved, but also the key to China’s energy conservation and emission reduction and high-quality economic growth, which represents green, low-carbon and sustainable economic development. Providing a holistic approach to solving the problem of pollution externality, environmental regulation is of great significance to achieve the long-term goal of green economic transformation and development. It is also compatible with the development concept of “innovation, coordination, green, openness and sharing”. The downside to this approach is that environmental regulation will increase the cost of high polluting enterprises [8] and increase the financing demand, which needs the support of a developed and sound financial system [9]. Under the background of increasingly serious environmental pollution, we should optimize the environmental regulation system, promote the full flow of financial resources within the region, give full play to the advantages of financial agglomeration, guide resources to green industries, and continuously improve green total factor productivity.

**Figure 1:**
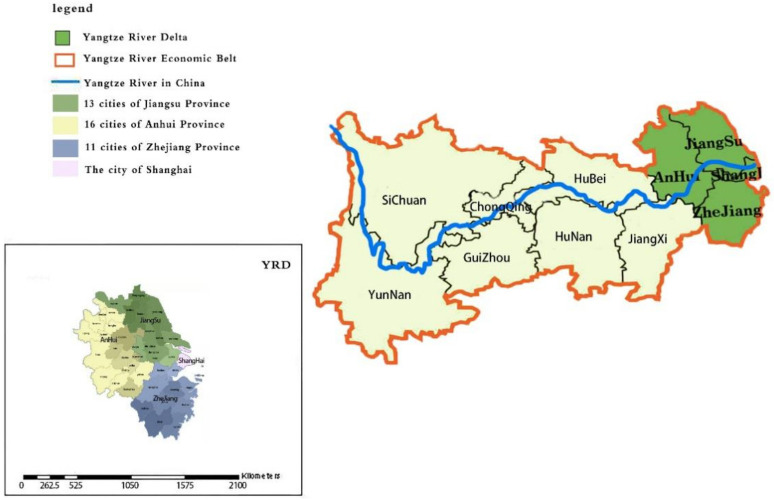
Geographic map of China where the Yangtze River Delta is the research Area.

The Yangtze River Economic Belt (YREB) is based on the Yangtze River includes 11 provinces and cities along the Yangtze River, including Shanghai, Jiangsu, Zhejiang, Anhui, Jiangxi, Hubei, Hunan, Chongqing, Sichuan, Guizhou, and Yunnan. The economic development of the Yangtze River Delta (YRD) has played a leading role in the development of other regions of the YREB. The areas of YRD include Shanghai, Jiangsu, Zhejiang, and Anhui, totaling 41 cities, as shown in Figure 1. The economic development of the YRD and the realization of green and sustainable development have played a leading and exemplary role in promoting the economic development of other regions in China. In the first half of 2021, the total gross domestic product (GDP) in the YRD exceeded US $2 trillion, accounting for about one-quarter of the China’s total GDP. It has become one of the regions with the highest level of economic development, the highest degree of openness and the strongest innovation ability in China. China’s State Council has issued the outline of the YRD integrated development plan, which is a programmatic document to guide the economic development of the YRD in the future. The YRD will place the protection of the ecological environment and the promotion of green economic development in an important position. It is very important to study the development of green economy in the YRD and explore the role of environmental regulation and financial development in promoting green economic development.

This article’s main goals are to (1) systematically analyze the impact of environmental regulation on the development of green economy, and test whether there is a nonlinear relationship between environmental regulation and the development of green economy; (2) construct the research framework of financial development, environmental regulation and green economic development, and test the impact of the collaborative mechanism of financial development and environmental regulation on the development of green economy. It is desired that suitable recommendations are made to decision-makers so that economic development can be green and sustainable.

The possible contributions of this paper are as follows: first, in the construction of core indicators, the entropy of index data is used to determine the weight of each indicator, and then the method of technology for order preference by similarity to ideal solution (TOPSIS) is used to comprehensively evaluate the financial development level and environmental regulation level of 41 prefecture-level cities in three provinces and one city in the YRD, to avoid the limitations of a single indicator. Second, in the measurement of green total factor productivity, the radial non-angle directional distance function and the global Malmquist Luenberger (GML) index are selected to measure the green total factor productivity including undesirable output, to avoid the possible unsolved problem of linear programming in cross period measurement and avoid the phenomenon of “passive” improvement of production efficiency and “technological regression”. Third, it improves the defects of previous studies using a single environmental regulation tool or financial development indicators, connects financial development and environmental regulation, and analyzes the impact of their interaction on Regional Green total factor productivity, which is innovative.

The rest of this paper is organized as follows. The second section (Section 2) presents a description of previous works of literature and tries to find the research gap of this study, the third section (Section 3) provides the description of the data as well as methodology, the fourth section (Section 4) presents the empirical results, and the fifth section (Section 5) provides the summary of this paper as well as some policy implications and suggestions regarding the achievement of Green economic development.

## 2. Literature Review

Green total factor productivity has become an important measure of green economic development. Scholars have presented some different views on environmental regulation and green total factor productivity for the past.

Cropper & Oate [10] put forward the cost hypothesis which explain the impact effect of environmental management production on enterprise total factor productivity and holds that strict environmental regulation policies will lead to the increase of enterprise production costs. In order to obtain higher profits or reduce costs, enterprises will give up green technology R & D, which will have an adverse impact on regional Green total factor productivity. Some researchers believe that environmental regulation is essentially the internalization of environmental pollution losses by enterprises, such internalization brings additional costs which restrict the level of technological innovation of enterprise. In practice, environmental regulation increases enterprise costs and management fees, which is not conducive to the improvement of total factor productivity, Greenstone [11], Wang et al. [12]. Chen [13] believe that for enterprises, both recycling and carbon emission capacity regulation will lead to a decline in profits, showing a negative effect. Jiang [14] found that industry environmental regulation has a negative impact on enterprise innovation performance. Tang [15] studied that environmental control regulations have had an adverse impact on small enterprises, state-owned enterprises, and enterprises in western and eastern China. Wang [16] studied that environmental regulation will indirectly restrict the improvement of industrial total factor productivity. The increase of environmental regulation intensity is not enough to make up for the resulting regulation compliance cost of enterprises, which will eventually reduce total factor productivity. Yang [17] suggested the contribution of eastern China is negative, which is related to the relatively low growth rate of environmental regulation intensity in this region.

Some researchers give another result. Porter [4] put forward Porter’s hypothesis theory, which highly agree with the innovation compensation effect of environmental regulation on green total factor productivity. Such scholars believe that appropriate environmental regulation can stimulate the “innovation compensation” effect, which do not only make up for the “compliance cost” of enterprises but also improve the productivity and competitiveness of enterprises and improve total factor productivity. Porter and Linde [18] explained that positive product innovation and process innovation can offset the cost pressure brought by environmental regulation in order to improve total factor productivity. Jaffe and Palmer [19] divided the Porter hypothesis into “weak Porter Hypothesis”, “strong Porter Hypothesis” and “narrow Porter Hypothesis”, which explained the mechanism of environmental regulation on green total factor productivity respectively. Empirical tests such as Guo [20], Zhang [21], Peng [22], Gong [23], Yu [24], Zhang [25] also show that the promulgation and implementation of local environmental regulations and standards have a positive effect on the total factor productivity of enterprises. The research conducted by Manello [26] based on the data of chemical industry enterprises in Germany and Italy shows that environmental regulation has an obvious effect in high pollution industries, especially in those areas with higher environmental regulation correspond to higher growth rates of green total factor productivity. Wang [27] shows that technological progress can significantly promote the level of green total factor productivity in surrounding areas.

In addition, some literature studies have found that environmental regulation promotes the improvement of green total factor productivity and has a superposition effect with regional economic development, foreign direct investment, fiscal decentralization, and other factors, Li [28], Song [29], Tang [30], Zhang [31], Tang [32]. Some literatures pay attention to the long-term and short-term effects of environmental regulation on green total factor productivity. It is founded that the effects of environmental regulation are periodic. In the short term, environmental regulation can promote the improvement of environmental efficiency, while in the long term, environmental regulation only improves energy efficiency but hinders labor productivity, which does not support the “strong” of Porter’s hypothesis edition, Yuan [33], Li and Chen [34].

In recent years, there are some literatures focusing on the nonlinear relationship between environmental regulation and green total factor productivity. Research shows that there will be a critical point between environmental regulation and green total factor productivity, and excessive environmental regulation will inhibit the promotion effect of green total factor productivity. Wang [35] based on the panel data of industrial sectors in OECD countries, and analyzes the strictness of environmental regulatory policies, and the Porter hypothesis is verified, that is, within a certain degree of strictness (less than 3.08), environmental policies have a positive impact on green productivity growth; When the environmental regulatory policy is strict to a certain extent, the impact will become adverse, because the cost-effectiveness of compliance is higher than the offset effect of innovation. These findings provide new empirical evidence for the powerful version of Porter’s hypothesis and some enlightenment for OECD countries to further promote green growth. Li [36] believes that the goal of environmental regulation is to achieve a win-win situation of economic growth and environmental quality at the same time. Environmental regulation can effectively promote industrial green transformation by improving green total factor productivity. Lei [37], Zhao [38] also found that environmental regulation has a nonlinear impact on green total factor productivity. The research results show that different types of environmental regulations have different effects on the green total factor productivity of different polluting industries, and the green total factor productivity of heavily polluting industries is in an inverted U shape [39]. The research results show that the impact of environmental regulation on green total factor productivity is an inverted U-shaped change that increases first and then decreases [40]. Li [41] studied the heterogeneity of the industry and considered that environmental regulation is a necessary means to achieve environmental benefits and improve the productivity of all green factors in the manufacturing industry. The environmental regulation of moderate pollution and light pollution industries is in a “U” shape, and there is no nonlinear relationship between the intensity of environmental regulation of light pollution industries.

Preceding literature mainly focused on the impact of environmental regulation on green total factor productivity. Under the forced effect of environmental regulation policy, enterprises urgently need to expand financing scale and improve technological innovation level to promote green economic transformation. Driven by this mechanism, financial resources will tend to be more concentrated in pollution-intensive enterprises and industrial sectors, Chang [42], Dong [43] are of the view that the credit funds of commercial banks will be inclined to heavily polluting industries, and high consumption and high pollution industries such as steel, coal and chemical industry have become the concentration area of credit resources. The mismatch of funds will aggravate the deterioration of the environment and is not conducive to the internal requirements of the development of a green economy. From the perspective of financial development, it is imperative to correctly and reasonably guide the flow of credit funds to ensure the normal development of a green economy. Zhou [44] discussed the direction and intensity of financial development affecting green total factor productivity. The results show that the increase in financial development will help to attract high-quality and low pollution foreign direct investment. There is a positive “U” relationship between environmental governance and green total factor productivity. Li [45] investigated the heterogeneous impact of financial development on green total factor productivity in 40 countries from 1991 to 2014 and confirmed that there is an inverted U-shaped relationship between financial development and green total factor productivity. Zhang [46] found that the green credit regulatory policy significantly improved the growth of green total factor productivity. At present, there is little literature to further analyze the mechanism of environmental regulation, financial development, and green total factor productivity. Scholars generally believe that financial development and environmental regulation will significantly promote the growth of green total factor productivity. There are differences in the effects of environmental regulation on improving green total factor productivity under different financing modes. The intensity of environmental regulation affects the efficiency of green development thereby affecting the allocation of financial resources. The enhancement of regional environmental regulation will cause a mismatch of financial resources.

Some scholars have studied the impact of the change of total factor productivity on the economy in the YRD. Zhang [47] believes that the total factor productivity in the YRD shows a downward trend, which is inseparable from the insufficient utilization of input and output. At present, there is little literature on financial development, environmental regulation, and the green total factor productivity in the YRD region. Based on the perspective of financial development, studying the innovation compensation effect of environmental regulation to improve green total factor productivity puts forward higher requirements for the development and improvement of China’s financial market system. The YRD is one of the most economically developed regions in China. Taking 41 cities in the YRD as research samples, this paper analyzes the impact of environmental regulation and green total factor productivity in regions with a high degree of economic development, as well as the advantages and disadvantages of financial forces in promoting green total factor productivity and improvement measures, which can supplement the shortcomings of the existing literature system.

## 3. Theoretical Mechanism and Research Design

### 3.1. Theoretical Mechanism

Porter’s hypothesis [4] holds that reasonable and strict environmental regulation policies will not only create “positive” externalities for social public services such as environmental quality and ecological protection but also encourage enterprises to invest more funds in green technology research and development in their production activities, resulting in positive externalities. In the long run, environmental regulation will directly affect enterprise resource investment and emission, force enterprises to carry out technological innovation, and the benefits will not only compensate the environmental costs paid by enterprises, but also carry out clean technologies and clean products that meet the requirements of green economy development through technological upgrading, complete industrial transformation and upgrading, and bring new profit growth points to enterprises.

Therefore, hypothesis 1 is put forward: the impact of environmental regulation on green total factor productivity is positive.

With the increasingly strict environmental regulation and the continuous improvement of the standards of environmental regulation by the government, the investment of enterprises in environmental transformation is increasing, the cost of R & D investment is too high, the risk is too large, and the income is uncertain. Enterprises have a strong motivation to give up technology R & D, the promotion effect of green total factor productivity of environmental policy is gradually not obvious, to inhibit the improvement of green total factor productivity.

Therefore, hypothesis 2 is proposed: with the strengthening of environmental regulation, environmental regulation will reduce green total factor productivity.

As a market means, under the constraints of environmental regulation, financial resource allocation will effectively guide and invest funds in the most money deficient industries through the scale, structure, and allocation efficiency of financial resource allocation, to realize the optimal allocation of resources in the whole society. However, in the process of green transformation of traditional industries, due to the tight time, some high polluting enterprises need to ensure that their enterprises meet the national environmental protection control requirements in a short time, to realize green transformation, and there is a huge financing demand in the short term. In the external financing structure, equity financing may dilute equity, so creditor’s rights financing is generally selected, and obtaining bank credit funds becomes the first choice. The short-term financing demand is easy to make the industrial sectors with high pollution and high energy consumption gather many credit funds, while other industrial sectors with low pollution and low energy consumption lack the attention of the banking sector, resulting in the short-term imbalance of the regional credit market and affecting the improvement effect of the regional green total factor production rate. On the other hand, bank loans usually prefer relatively mature enterprises, while most innovative enterprises in the green industry have just been established or are still in the primary development stage, the technology R & D cycle is relatively long, there are medium and long-term financing needs, the risk is uncertain, and their objective conditions are difficult to obtain bank trust and financial support, resulting in limited financing for these enterprises, It is difficult to realize technological innovation, which will also restrict the promotion effect of green total factor productivity to a certain extent.

Therefore, hypothesis 3 is put forward: the strengthening of environmental regulation will lead to more credit resources inclined to pollute enterprises, and it will also make it difficult for innovative enterprises in the green industry to obtain financial support in time, resulting in the reduction of green development efficiency.

### 3.2. Model Construction

Based on the new economic growth theory and the construction method of Miller and Upadhyay [48], this paper analyzes the effects of various factors on total factor productivity. Firstly, to keep the analysis simple, we adopt the Cobb–Douglas production function. The production function adopted is as follows:

(1) $$Y=A\left(.\right)\;\times \;f\left(K,L\right)$$

It is assumed that green total factor productivity is not only affected by environmental regulation but also depends on the level of financial development. Y represents the actual output level, $A\left(.\right)$ represents the green total factor productivity considering energy input and undesirable output, K represents capital input and L represents labor input.

Based on the research object of this paper, assuming that $A\left(.\right)$ is a function including influencing factors, the expression is embodied as:

(2) $$Y=A\left(ER\right)\;\times \;f\left(K,L\right)\;=A_{0}\times ER^{\alpha }\times f\left(K,L\right)$$

ER represents the level of environmental regulation. Considering time variable t and urban variable i, deform Equation (2) into Equation (3), $A_{0}$ is the initial value.

(3) $$Y_{i,t}=AER_{i,t}\times f\left(K_{i,t},L_{i,t}\right)\;=A_{i,0}\times ER_{i,t}^{\alpha i}\times f\left(K_{i,t},L_{i.t}\right)$$

$A_{i,0}\;$is the initial value, $ER_{i,t}$ is the environmental regulation variable of the city at time t, αi is the influence coefficient of corresponding factors on the technical level.

According to the characteristics of total factor productivity and Cobb Douglas production function, divide both sides of Equation (3) by $f\left(K_{i,t},L_{i,t}\right)$, $GTFP_{i,t}$ is the total factor productivity variable of the city at time t.

(4) $$GTFP_{i,t}=A_{i,t}=\frac{Y_{i,t}}{f\left(K_{i,t},L_{i.t}\right)}=\frac{A_{i,0}\times ER_{i,t}^{\alpha i}\times f\left(K_{i,t},L_{i.t}\right)\;}{f\left(K_{i,t},L_{i,t}\right)}=A_{i,0}\times ER_{i,t}^{\alpha i}$$

Take logarithms from both sides of Equation (4) and add a constant term and error term to obtain Equation (5) to test hypothesis 1.

(5) $$InGTFP_{i,t}=InA_{i,0}+\alpha iInER_{i,t}\;InGTFP_{i,t}=\mu_{1}+\alpha iInER_{i,t}+\varepsilon_{i,t}$$

Based on the Equation (5), add the quadratic term of environmental regulation. As shown in Equation (6), analyze whether there is a nonlinear relationship between the environmental regulation and the total factor productivity to test hypothesis 2.

(6) $$InGTFP_{i,t}=\mu_{1}+\alpha iInER_{i,t}+\beta i{\left(InER_{i,t}\right)}^{2}+\varepsilon_{i,t}$$

where αi, βi is the influence coefficient of factors on the technical level.

Separating the interaction between financial development and environmental regulation, it is biased to study their impact on green total factor productivity respectively. Based on the Equation (5), the interaction term between financial development and environmental regulation is introduced to test hypothesis 3, as shown in Equations (7) and (8).

(7) $$InGTFP_{i,t}=\mu_{1}+\alpha iInER_{i,t}+\beta iInFD_{i,t}+\varepsilon_{i,t}$$

(8) $$InGTFP_{i,t}=\mu_{1}+\alpha iInER_{i,t}+\beta iInFD_{i,t}+\gamma iIn(FD_{i,t}\times ER_{i,t})+\varepsilon_{i,t}$$

Equation (7) tests the single impact effect of environmental regulation and financial development on green total factor productivity, and Equation (8) tests the comprehensive impact of the interaction effect of environmental regulation and financial development on green total factor productivity, αi, βi and γi are the influence coefficient of relevant elements of Equations (7) and (8) on the technical level respectively.

In addition, based on Equations (5)–(8), add control variables such as government intervention, foreign investment utilization level and trade openness, and study the mechanism of the impact of other factors on green total factor productivity to obtain Equations (9)–(12), in which $Controls_{i,t}$ represents the control variable, $\delta_{i,t}$ is the coefficient of the control variable.

(9) $$InGTFP_{i,t}=\mu_{1}+\alpha iInER_{i,t}+\sum \delta_{i,t}Controls_{i,t}+\varepsilon_{i,t}$$

(10) $$InGTFP_{i,t}=\mu_{1}+\alpha iInER_{i,t}+\beta i{\left(InER_{i,t}\right)}^{2}+\sum \delta_{i,t}Controls_{i,t}+\varepsilon_{i,t}$$

(11) $$InGTFP_{i,t}=\mu_{1}+\alpha iInER_{i,t}+\beta iInFD_{i,t}+\sum \delta_{i,t}Controls_{i,t}+\varepsilon_{i,t}$$

(12) $$InGTFP_{i,t}=\mu_{1}+\alpha iInER_{i,t}+\beta iInFD_{i,t}+\gamma iIn\left(FD_{i,t}\times ER_{i,t}\right)\;+\sum \delta_{i,t}Controls_{i,t}+\varepsilon_{i,t}$$

In conclusion, we will divide the empirical analysis into two parts. First, without considering the control variables, we will investigate the primary and secondary effects of environmental regulation and green total factor productivity, and the relationship between the interaction between financial development and environmental regulation and green total factor productivity. Second, we add control variables to investigate the impact of control variables on green total factor productivity and compare whether the control variables will offset the primary effect, secondary effect and interaction effect.

### 3.3. Index Selection

#### 3.3.1. The Explained Variable

The explained variable is green total factor productivity, which is used to measure the level of green economic development.

In the process of calculating total factor productivity, some scholars have taken resources, environment, and other factors into account. Pittman [49] and others put pollution in the production function as undesirable output for the first time. Ming [50], Wang [27] included pollution emissions in undesirable output to study its role in economic growth. By combining the traditional Malmquist index with the directional distance function (DDF), the desirable and undesirable output are included in the study of total factor productivity at the same time. Pastor [51] proposed a global measurement technology based on the overall situation, taking all measurement periods as the cutting-edge reference benchmark. Oh [52] considered the desirable output (benefits increase) and undesirable output (environmental pollution) in the production process, applied the global idea to Malmquist-Luenberger (ML), established the global production possibility set and directional distance function, focused on analyzing the cumulative change of production efficiency and proposed the GML index.

This paper comprehensively considers the constraints of resources, energy, and environment, and selects the radial non-angle directional distance function and GML index to calculate, to ensure that the final production possibility concentration can include desirable and undesirable output. Based on Chung [53], a production possibility set including desirable output and undesirable output is constructed. Each city in 41 cities in the YRD is used as a decision-making unit. Each decision-making unit adopts R inputs, and Q desirable outputs and H undesirable outputs can be obtained. Assuming that X, Y, and B are inputs, desirable outputs, and undesirable outputs respectively, $g_{x},g_{y},g_{b}$ are the vector of input, desirable output and undesirable output, *β* is the degree of inefficiency, and the directional distance function represents the maximum expansion multiple of output y, b in the direction of g under a certain input X. The value is inversely proportional to the production efficiency.

(13) $$\vec{D_{0}^{t}}\left(\;X_{k_{0}}^{t},Y_{k_{0}}^{t}B_{k_{0}}^{t}g_{x},g_{y},-g_{b}\right)=max\;\beta$$

Based on constructing the directional distance function and referring to the method of Oh [30], the global benchmark technology $P^{G}$ is constructed, that is $P^{G}=P^{1}\cup P^{2}\cup \cdots P^{T}$.

The GML index expression is:

(14) $$GML^{t,t+1\;}\left(x^{t},y^{t},b^{t},x^{t+1},y^{t+1},b^{t+1}\right)=\frac{1+D^{G}\left(x^{t},y^{t},b^{t}\right)}{1+D^{G}\left(x^{t+1},y^{t+1},b^{t+1}\right)}$$

The GML index is divided into two parts: the change from the forefront of best practice (BPC), and the change of technical efficiency (EC).

(15) $$GML^{t,t+1\;}\left(x^{t},y^{t},b^{t},x^{t+1},y^{t+1},b^{t+1}\right)=\frac{1+D^{G}\left(x^{t},y^{t},b^{t}\right)}{1+D^{G}\left(x^{t+1},y^{t+1},b^{t+1}\right)}\;=\frac{1+D^{t}\left(x^{t},y^{t},b^{t}\right)}{1+D^{t+1}\left(x^{t+1},y^{t+1},b^{t+1}\right)}\times \left[\frac{\frac{\left(1+D^{G}\left(x^{t},y^{t},b^{t}\right)\right)}{\left(1+D^{t}\left(x^{t},y^{t},b^{t}\right)\right)}}{\frac{\left(1+D^{G}\left(x^{t+1},y^{t+1},b^{t+1}\right)\right)}{\left(1+D^{t+1}\left(x^{t+1},y^{t+1},b^{t+1}\right)\right)}}\right]\;=\left[TE^{t+1}/TE^{t}\right]\times \left[BPG_{t+1}^{t,t+1}/BPG_{t}^{t,t+1}\right]=EC^{t,t+1}\times BPC^{t,t+1}$$

where $BPC^{t,t+1}$ indicates the change from the forefront of best practice, from the period of the t to t+1. That is the judgment standard for technological progress. When the index is greater than 1, it indicates technological progress, and when the index is less than 1, it indicates technological regression. $EC^{t,t+1}$ indicates the change of technical efficiency from the time t to t+1. When the index is greater than 1, it indicates that it exceeds the technical frontier of the same period. When the index is less than 1, it indicates that there is a lag compared with the technological frontier in the same period.

When calculating the green total factor productivity index, the input index of this paper selects capital, labor, energy and water resources, the output index selects the actual GDP as the desirable output, and the industrial wastewater and waste gas emission is the undesirable output.

The capital input is expressed by the actual capital stock according to the calculation formula of the perpetual inventory method.

(16) $$K_{it}=I_{it}+\left(1-\delta_{it}\right)\times K_{it-1}$$

where $K_{it}$, $K_{it-1}$ represents the actual capital stock of city i in year t and year t−1, $I_{it}$ refers to the actual fixed asset investment of city i in the year t, since the fixed asset investment price index of each city cannot be obtained, this paper uses the fixed asset investment price index of each city’s province to replace the fixed asset investment price index of each city in the calculation, with 2000 as the base period, The total investment in fixed assets of each city is reduced to obtain the actual investment in fixed assets of the city i in the year t.

Labor input is expressed by employees of the whole society, and the unit is 10,000 people.

Energy input is expressed by the power consumption of the whole society, in kwh.

The input of water resources is expressed by the total amount of regional water resources, with the unit of 100 million cubic meters.

Desirable output, expressed in real GDP, with 2000 as the base period, unit: 100 million yuan.

Undesirable output, using industrial wastewater discharge, unit: 10,000 tons; Industrial sulfur dioxide emission, unit ton; Industrial smoke (powder) dust emission, unit: ton.

All the data are from the official websites of China Statistics Bureau, Shanghai Statistics Bureau, Jiangsu Statistics Bureau, Zhejiang Statistics Bureau and Anhui Statistics Bureau.

#### 3.3.2. Explanatory Variable

Through combing the existing studies, it is found that most single indicators only focus on a specific dimension of environmental regulation or financial development and cannot comprehensively measure the degree of environmental regulation and financial development. This paper uses the entropy of index data to determine the weight of each index and then uses the TOPSIS method to evaluate the relevant data, to obtain the comprehensive index of environmental regulation and financial development.

1. Entropy weight method to determine the weight

The first is the homogenization of indicators

(17) $$\mathrm{Positive}\;\mathrm{index}:\;Y_{ij}=\frac{X_{ij}}{max\left(X_{ij}\right)}$$

(18) $$\mathrm{Negative}\;\mathrm{index}:\;Y_{ij}=\frac{min\left(X_{ij}\right)}{X_{ij}}$$

where $X_{ij}$ is the original data of the *j*-th index of the *i*-th City, $Y_{ij}\;$represents the measurement value of the *j*-th index of the *i*-th city after standardization (*i* = 1, 2, 3, ·, *m*; *j* = 1, 2, 3, ·, *n*).

Next, calculate the proportion of the *i*-th city in the *j*-th index.

(19) $$P_{ij}=\frac{Y_{ij}}{\sum \begin{array}{c} m\\ i=1 \end{array}Y_{ij}}$$

Then we calculate the entropy of the *j*-th index.

(20) $$E_{j}=-k\overset{m}{\underset{i=1}{\sum }}P_{ij}.In\left(P_{ij}\right)$$

(21) $$k=\frac{1}{In\left(m\right)}$$

Finally, the weight of the *j*-th index is calculated.

(22) $$W_{j}=\frac{1-E_{j}}{\sum \begin{array}{c} n\\ j=1 \end{array}\left(1-E_{j}\right)}$$

0$\;\leq W_{j}\leq 1,\;$and $\sum \begin{array}{c} m\\ j=1 \end{array}W_{j}=1$.

2. Use the TOPSIS method to evaluate relevant data

TOPSIS is an analysis method suitable for the comparison and selection of multiple indexes and schemes, which is used as the standard to evaluate the advantages and disadvantages of the scheme [54,55].

Firstly, the normalized decision matrix and weight vector are used to form a weighted decision matrix.

(23) $$R={\left(r_{ij}\right)}_{m.n},r_{ij}=W_{j}.Y_{ij}\left(i=1,2,3\cdots ,m;j=1,2,3,\cdots ,n\right)$$

R = $\left[\begin{array}{cccc} W_{1}Y_{11} & W_{2}Y_{12} & \cdots & W_{n}Y_{1n}\\ W_{1}Y_{21} & W_{2}Y_{22} & \cdots & w_{n}Y_{2n}\\ & \vdots \;\vdots \;\ddots & \vdots & \\ W_{1}Y_{2m} & W_{2}Y_{2m} & \cdots & W_{n}Y_{mn} \end{array}\right]$, and,$\;W_{j}=\left[\begin{array}{cccc} W_{1} & 0 & 0 & 0\\ 0 & W_{2} & 0 & 0\\ 0 & 0 & \ddots & 0\\ 0 & 0 & 0 & W_{n} \end{array}\right]$

Then, the ideal solution $S^{+}$ and negative ideal solution $S^{-},\;$and the vectors composed of the maximum and minimum values of each column are determined.

(24) $$S_{j}^{+}=max\left(r_{1j},r_{2j},\cdots ,R_{nj}\right)$$

(25) $$S_{j}^{-}=min\left(r_{1j},r_{2j},\cdots ,r_{nj}\right)$$

Third, determine the distance between the evaluation object and the ideal solution and the negative ideal solution.

(26) $$D_{i}^{+}=\sqrt{\overset{n}{\underset{j=1}{\sum }}{\left(S_{i}^{+}-r_{ij}\right)}^{2}}$$

(27) $$D_{i}^{-}=\sqrt{\overset{n}{\underset{j=1}{\sum }}{\left(S_{i}^{-}-r_{ij}\right)}^{2}}$$

Finally, determine the proximity $C_{i}$ between the evaluated object and the optimal scheme.

(28) $$C_{i}=\frac{D_{i}^{-}}{D_{i}^{+}+D_{i}^{-}}$$

That is, the comprehensive evaluation value of the degree of financial development and environmental regulation of each city. The greater the value, the higher the degree of environmental regulation and financial development.

Environmental regulation is an important part of social regulation. The government can regulate enterprise economic activities by formulating corresponding policies and measures, and it also can reduce the negative externality of environmental pollution caused by enterprise production activities, realizing the coordinated development of environment and economy. It is often difficult to obtain more accurate reference variables of environmental regulation. Some researchers took the proportion of total investment in industrial pollution control in the main business cost or industrial added value of Enterprises above designated size as the intensity index of environmental regulation, which is considered to underestimate the actual level of local environmental regulation. They constructed a comprehensive index based on pollutants removal rate to measure the local environmental regulation, including some index such as removal of industrial dust and sulfur dioxide. In this paper, the environmental regulation index system uses three indicators: industrial wastewater emission, industrial sulfur dioxide emission and industrial smoke (powder) dust emission [16,17].

FD represents the corresponding proxies of FD, including the balance of deposits and loans per capita (the sum of deposits and loans of regional financial institutions and the proportion of the total population), the conversion rate of deposits (the ratio of deposits to loans) and the financial scale (the sum of deposits and loans of regional financial institutions accounts for the proportion of GDP), [6,9], all of which are used to measure the financial scale, structure and efficiency. The financial market includes not only the indirect financing market formed by financing with commercial banks as credit intermediaries, but also the stock market, bond market and other direct financing markets that do not need financial intermediaries. However, based on the limitations of prefecture level data, this paper cannot completely collect the basic data such as stock financing amount and bond financing amount at prefecture level, Therefore, when analyzing financial development indicators, only the basic data of banks are selected. The specific calculation method is as shown above. Finally, the environmental regulation indicators and financial development indicators are calculated. The relevant data are from the official websites of the China Statistics Bureau, Shanghai, Jiangsu, Zhejiang, and Anhui Statistics Bureaus.

#### 3.3.3. Control Variables

This paper selects the proportion of fiscal expenditure in GDP, the proportion of foreign investment in GDP and the proportion of total import and export trade in GDP to measure the impact of government financial intervention, foreign capital utilization and opening to the outside world on green total factor productivity in the YRD. When calculating the proportion of foreign investment in GDP and the proportion of total import and export trade in GDP. Based on the annual average exchange rate, the unit of US dollar is adjusted to the unit of RMB. The relevant data are from the official websites of the China Statistics Bureau, Shanghai, Jiangsu, Zhejiang, and Anhui Statistics Bureaus. The variables involved in this paper are shown in Table 1.

## 4. Result from Analysis

### 4.1. Spatial Difference Analysis of Green Total Factor Productivity in YRD

**Figure 2:**
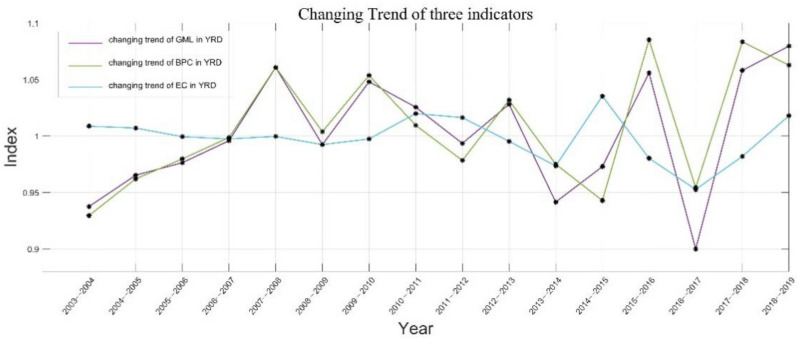
Changing trend of GML, BPC and EC in the YRD from 2003 to 2019.

**Figure 3:**
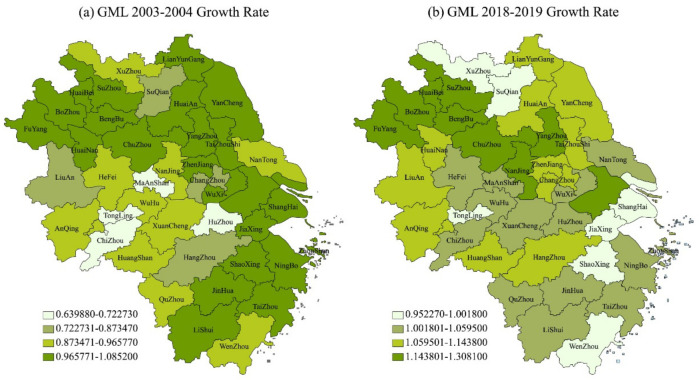
GML Growth rate in YRD (**a**) from 2003 to 2004, and (**b**) from 2018 to 2019.

**Figure 4:**
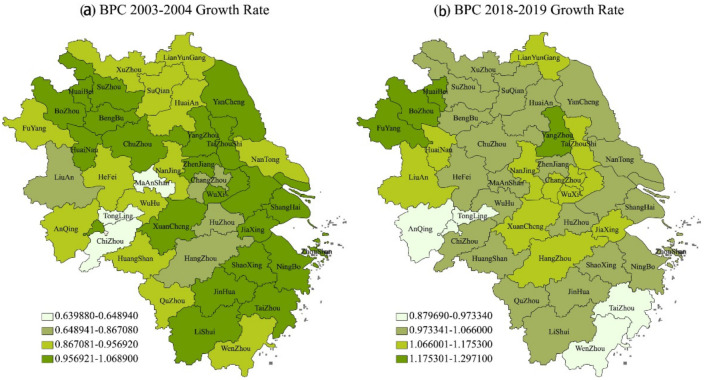
Growth rate of BPC in YRD (**a**) from 2003 to 2004, and (**b**)from 2018 to 2019.

**Figure 5:**
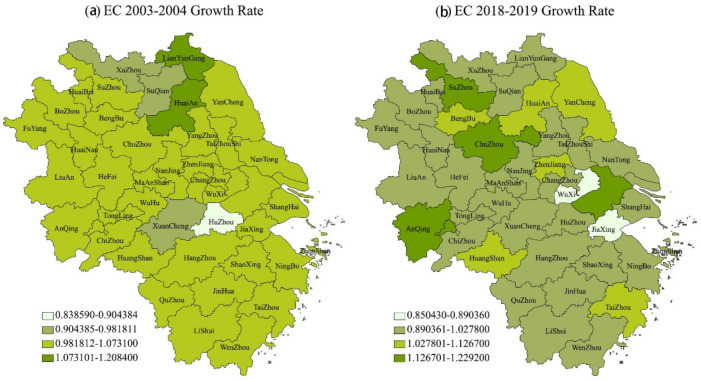
Growth rate of EC in the YRD (**a**) from 2003 to 2004, and (**b**) from 2018 to 2019.

Table 2, Table 3 and Table 4 respectively show the specific results of the provincial and annual GML and its decomposition indicators, technical progress and technical efficiency relative to the growth rate of the previous year in 41 cities in the YRD from 2003 to 2019. Figure 2 shows the distribution of GML, BPC and EC in the YRD from 2003 to 2019. Figure 2, Figure 3, Figure 4 and Figure 5 show the spatial distribution of GML growth rate, BPC growth rate and EC growth rate in the YRD, respectively.

From the overall average of the three growth rates, the annual average GML, BPC and EC in the YRD are 1.001955, 1.006976, and 0.998458, respectively. The average values of the first two growth rates are greater than 1, indicating that the green total factor productivity and technological step-by-step growth rate in the YRD are generally effective, while the average growth rate of technological efficiency is less than 1, The weak effectiveness of technical efficiency also means that it lags slightly behind the benchmark technology frontier in the same period. From the overall average of the three growth rates, the annual average growth rates of green total factor productivity GML, BPC and EC in the YRD are 1.001955, 1.006976 and 0.998458, respectively. The average values of the first two growth rates are greater than 1, indicating that the green total factor productivity and technological step-by-step growth rate in the YRD are generally effective, while the average growth rate of technological efficiency is less than 1, The weak effectiveness of technical efficiency also means that it lags slightly behind the benchmark technology frontier in the same period.

From the overall change trend of the growth rates of BPC and EC, as shown in Table 3 and Table 4, the average annual growth rates of the two decomposition indicators also showed a slow fluctuating upward trend from 2003 to 2019, and the annual fluctuation range of BPC was greater than that of EC, except that the average annual growth rate of EC was higher than that of BPC in 2003–2007, 2011–2012 and 2014–2015, In other years, the average annual growth rate of BPC exceeds that of EC. This shows that the YRD mainly depends on the improvement of technological progress to improve the growth level of regional green total factor productivity and realize the green transformation of the regional economy. In terms of provinces, Shanghai has the highest average annual growth rate of BPC, with an average annual growth rate of 1.024638 from 2003 to 2019, significantly leading. Zhejiang Province ranks second with 1.014231, and Jiangsu Province ranks third with 1.004858. Although Anhui Province ranks last, it also reaches 1.002605. From the perspective of EC growth, the order of growth rates from 2003 to 2019 is Anhui Province, Jiangsu Province, Shanghai city and Zhejiang Province. The growth rates of each province are close, and the average annual growth rates are 1.004294, 1.000103, 1 and 0.998458 respectively. Wet can find that different provinces have different paths to achieve green economic transformation. Shanghai, Zhejiang, and other economically active regions may take technological progress as the realization path to improve green development in their own regions, while Anhui and Jiangsu provinces may need to improve technological efficiency to improve green total factor productivity. In terms of cities, 33 of the 41 cities in the YRD have a technological progress efficiency growth rate of more than 1. Quzhou, Shaoxing and Shanghai rank the top three in terms of annual growth rate, and Huainan, Ma’anshan and Tongling in Anhui Province rank the last, which is basically consistent with the ranking of the provinces. The results of EC’s growth rate show that among the 41 cities in the YRD, 23 cities have a technical efficiency growth rate of more than 1. Bengbu, Lianyungang and Nanjing rank first in the average annual growth rate, and Jiaxing, Huzhou and Suqian rank last, which is consistent with the ranking of provinces. The analysis results by provinces and cities show that technological progress and technological efficiency will have an impact on the development of regional green transformation, but there are regional differences. From the decomposition index of all green elements in China’s most economically developed regions, the YRD depends more on improving the level of technological progress and increasing the level of environmental governance, Promote the improvement of the green development level of the regional economy. Therefore, the government’s optimal environmental regulation intensity should be implemented, and we should not only pay attention to technological innovation, but also pay attention to technological efficiency [41].

### 4.2. Empirical Regression Analysis

In this paper, the System GMM estimation method is used to estimate the set model, and the specific results are shown in Table 5 and Table 6. The results show that AR (1) of all regression models is less than 0.05 and AR (2) are greater than 0.05, indicating that there is no second-order sequence-related assumption for the error terms of all regression equations, and Hansen test is greater than 0.1, indicating that the instrumental variables are effective. Therefore, it can be determined that the model setting in this paper is reasonable and effective.

#### 4.2.1. Empirical Result Analysis of Single Variable

The test without considering control variables is shown in Table 5. The test results of Equation (5) show that without considering other factors, there is a significant positive correlation between environmental regulation and green total factor productivity, and the regression coefficient is 0.312018, which is significant at the level of 1%, indicating that an increase of one unit in the intensity of environmental regulation will significantly increase the green total factor productivity in the YRD by 0.312018, which is consistent with Porter’s theory, Hypothesis 1 is verified [4,18,19,20,21,22,23,24,25,26,27]. The YRD is one of the most economically developed regions in China. At present, the construction of the YRD ecological green integrated development demonstration area is focusing on a high level, striving to promote the ecological environment protection and green development of the demonstration area, and appropriately strengthening environmental regulation can promote the regional economy to a deeper and higher level of green coordinated development.

Equation (6) adds the secondary term of environmental regulation to Equation (5) to test whether there is a nonlinear relationship between environmental regulation and green total factor productivity [35,36,37,38,39,40,41]. The results show that the primary term coefficient is positive, and the secondary term is negative, and both pass the 1% significance level test, indicating that environmental regulation can promote the development of regional green economy. With the strengthening of environmental regulation, it will inhibit the growth of green total factor productivity, showing an inverted U-shaped change, which verifies hypothesis 2. The calculation shows that the inflection point of environmental regulation is 0.6157, that is, the environmental regulation is on the left side of 0.6157. Increasing environmental regulation can effectively promote the growth of green total factor productivity in the YRD. However, if it is too harsh, pursue the absolute pollution control effect, continuously increase the pollution control burden of enterprises, and the level of environmental regulation exceeds the inflection point. It will restrict the promotion effect of green total factor productivity and is not conducive to the green transformation of regional economic development. Although the degree of industrialization in the YRD is high, there is also a phenomenon of regional development imbalance, which is not only reflected in the total industrial output value but also the control of pollutant emission in the process of industrialization. The above analysis of green total factor productivity also confirms this point. There are great differences in green production efficiency in different regions of the YRD, and there is also an imbalance in different pollution emission control in the same region. Therefore, environmental regulation is the key factor of green development in the YRD, but it needs appropriate regulation to avoid negative restrictive effects.

Equations (7) and (8) are used to test the impact of financial development and environmental regulation on green total factor productivity, and the impact of the interaction between financial development and environmental regulation on green total factor productivity. The results of Equation (7) show that the effects of financial development and environmental regulation on green total factor productivity are positive, and both have passed the 1% test, and the regression coefficients are 0.226589 and 0.304650 respectively, indicating that without considering other factors, financial development and environmental regulation will play a role in promoting green economic transformation. The results of Equation (8) show that the cross term coefficient of financial development and environmental regulation is positive, which is significant at the 1% level, and the regression coefficient is much greater than the coefficients of the two explanatory variables of financial development and environmental regulation, showing a strong interactive superposition effect, indicating that the interactive effect of the two has a great impact on the growth of green total factor productivity, which is contrary to hypothesis 3. As the most economically developed region in China, in the first half of 2021, the GDP of three provinces and one city in the YRD, Shanghai, Jiangsu, Zhejiang, and Anhui exceeded 13 trillion, accounting for about one-quarter of China. The region has established a financial cooperation mechanism, a sound financial system arrangement and organizational framework, and a cross-regional financial cooperation platform Continuously improves the efficiency of resource allocation. Therefore, whether in the green transformation of traditional industrial sectors or at the initial stage of establishment, innovative green industrial enterprises in this region can raise funds through a variety of financing channels. It is difficult to see the decline of total factor productivity due to the lack of financing funds stated in hypothesis 3, which is the main reason contrary to hypothesis 3. If we can make full use of the power of finance, give full play to the capital accumulation effect of finance, guide the flow of credit funds to low pollution industries and enterprises in the region, support green technology innovation and promote technological progress, we can eliminate the financing difficulties of enterprises caused by environmental regulation, give better play to their transmission and superposition role, and promote the green growth of the YRD.

#### 4.2.2. Analysis of Empirical Results by Adding Control Variables

The regression test results considering the control variables are shown in Table 6, and the conclusions are consistent with the below. Equation (9) shows that environmental regulation will positively promote the improvement of green total factor productivity. It has passed the test with a significance level of 1% and verified hypothesis 1. At the same time, increasing government intervention, implementing trade opening, and increasing foreign trade business will improve green total factor productivity, but the introduction of foreign capital will restrict the development level of a green economy.

Equation (10) shows that the impact of environmental regulation on green total factor productivity is inverted U-shaped. After calculation, the inflection point is 0.5034, which is lower than the data of Equation (6). The possible reason is that the control variable fiscal expenditure has a significant negative impact effect in this test, which indirectly weakens the positive effect of environmental regulation on economic green development. At the same time, it also shows that on the premise of considering the nonlinear impact, we should try to prevent the negative impact of environmental regulation in advance and give appropriate play to its role in promoting the development of a green economy.

The results of Equations (11) and (12) are consistent with the above, which verify the superposition effect of financial power on environmental regulation green economic development.

The results of control variables show that the impact of fiscal expenditure on green total factor productivity is significantly negatively correlated, that is, the government’s fiscal intervention can be reduced under the joint force of financial development and environmental regulation. The regression coefficient of the proportion of foreign investment shows a negative effect at the significance level of 1%, indicating that the introduction of foreign capital should be appropriately controlled, the flow of foreign capital to green industries should be guided, and the use efficiency of foreign capital should be improved, so as to restrain the hindering effect of foreign capital introduction on the improvement of green total factor productivity. The regression coefficient of the degree of opening to the outside world is significantly positive, which further confirms the importance of opening to the outside world and international trade development.

## 5. Research Conclusions and Countermeasures

### 5.1. Research Conclusions

Taking 41 cities in the YRD of China as the research sample, this paper selects the relevant input-output data from 2003 to 2019, selects the radial non-angle directional distance function and GML index to measure the green total factor production rate and its decomposition index of 41 cities in the YRD including undesirable output, This paper analyzes the improvement and regional distribution characteristics of green total factor productivity in China’s most economically developed YRD, and uses the systematic GMM estimation method to explore the impact of environmental regulation on the growth effect of green total factor productivity in the YRD, This paper studies whether there is a superposition effect of financial power on environmental regulation-Green total factor productivity.

Firstly, the growth rates of green total factor productivity GML and technological progress BPC in the YRD show a slow fluctuating upward trend, and the growth rates rise and fall between different years. The YRD depends more on improving the level of technological progress, increasing the level of environmental governance and promoting the improvement of regional economic green development level.

Secondly, there is a nonlinear relationship between environmental regulation and green total factor productivity Too strict environmental regulation will inhibit the growth of green total factor productivity. By adding control variables, the inflection point of environmental regulation is 0.5034, which is lower than that without control variables.

Thirdly, financial development and environmental regulation will play a role in promoting green economic transformation, and the cross term test of financial development and environmental regulation shows a strong interactive superposition effect, indicating that the interactive effect of the two has a great impact on the growth of green total factor productivity, which is consistent with the established financial cooperation mechanism in the YRD.

Fourthly, the impact of fiscal expenditure on green total factor productivity is significantly negatively correlated. Government intervention should be reduced, and the proportion of foreign investment shows a negative effect. The introduction amount of foreign capital should be appropriately controlled to guide foreign capital to green industries and improve the utilization efficiency of foreign capital. The strengthening of opening to the outside world is conducive to promoting the improvement of green total factor productivity.

Furthermore, some literature conclusions show that, the effects of technological change induced by environmental regulation is weaken due to the region’s relatively lower growth rate in environmental regulation intensity, the larger the exogenous technological change, the less the technological efficiency improves Yang [17]. Since China’s environmental regulatory policies were mainly command-and-control policies and pollutant drainage fee policies, which tended to focus on the terminal treatment of pollutant emissions, it is impossible to attain the innovative incentive effect. We have known that the development of green economy in the YRD mainly depends on technological progress, but environmental technological progress caused by environmental regulation may have a non-neutral impact on factor input and output [56]. With the perfect financial institutional arrangements and cross-regional financial cooperation platforms in YRD, it is concluded that environmental regulation can increase the development of Green Economy by encouraging the development of the financial sector.

### 5.2. Countermeasures and Suggestions

#### 5.2.1. Pay Attention to the Strength of Environmental Regulation and Prevent over-Correction

The realization of green economy transformation depends on the implementation of strict environmental regulation policies, but we should pay attention to the strength of environmental regulation. When introducing or formulating environmental governance policies, we should focus on sustainable economic and social development and comprehensively evaluate the economic and social effects of environmental policies, rather than simply emphasizing the short-term governance objectives of an environmental regulation policy. At the same time, we need to focus on the sustainability of environmental policies. In the later stage of the implementation of environmental policies, we should adjust measures to time, timely revise and improve the original policies, promote policy deepening, and ensure the coherence of environmental policies and the sustainability of benign economic and social effects. In the specific implementation process, a series of evaluation and early warning mechanisms should be established to monitor the intensity of environmental regulation in real-time to ensure the effectiveness of the implementation of environmental regulation.

#### 5.2.2. Increase the Guidance of Credit Funds and Deepen the Reform of the Financial System

Build a multi-level financial market structure, optimize the overall layout of regional finance, develop green credit and green bond market, open up the Initial Public Offering (IPO) channel of green enterprises, increase the guidance of credit funds, and enhance the ability of financial services for the transformation and development of a green economy. Based on the improvement of environmental regulation, there may be problems such as difficult and expensive financing. We should strengthen financial reform, encourage more high-quality private enterprises to finance green technology innovation funds through the stock market and green financial product market and alleviate the adverse impact of credit mismatch on the financing of green technology innovation funds of private enterprises. At the same time, we also need to control financial risks, improve the risk dispersion mechanism of green technology innovation by developing green innovation insurance products or establishing green innovation R&D risk fund to encourage more R&D subjects to carry out green technology innovation activities and promote the green transformation and development of a regional economy.

#### 5.2.3. The Government Intervenes Moderately to Strengthen the Guidance of Foreign Investment

Preferential fiscal and tax policies need to be introduced to encourage local enterprises to strengthen environmental protection technological innovation, but they should be appropriate and give full play to the role of the market in resource allocation. At the same time, we will strengthen the screening of foreign direct investment enterprises, strictly restrict the access of foreign direct investment with high pollution, high energy consumption and low technology content, encourage and guide foreign direct investment into the fields of environmental protection, clean energy, and high-tech industries, improve the green technology innovation level of domestic enterprises and promote the green development level of the regional economy.

There are some limitations that require further research. The indicators to measure environmental regulation include the proportion of total investment in pollution control in GDP. However, since we study the sample of Chinese cities, the indicators of some cities have not been fully published in some years, resulting in the lack of sample data. Empirical analysis with the missing data will reduce the accuracy of the results. Therefore, the robustness test cannot be conducted at present, which is also the limitation of our research. In the future, we will conduct more in-depth research on environmental regulation, financial development and green total factor productivity. We will use China’s provincial data as a sample to study the impact of environmental regulation on China’s green total factor productivity and regional differences, Since the provincial level data include the proportion of total pollution investment in GDP, we will verify whether the indicators of different environmental regulations can get the same empirical results.

## Figures and Tables

**Table 1:** Description of indicators.

Variable	Index	Computing Method
Environmental regulation	ER	Select three indicators: industrial wastewater emission, industrial sulfur dioxide emission and industrial smoke (powder) dust emission, determine the weight of each indicator by using the entropy of the indicator data, and then evaluate the relevant data by TOPSIS method.
Financial development	FD	Select three indicators: per capita deposit and loan balance, deposit conversion rate and financial scale, determine the weight of each indicator by using the entropy of the indicator data, and then evaluate the relevant data by TOPSIS method.
Financial intervention	FI	The proportion of fiscal expenditure in GDP
Foreign capital utilization	FDI	Measured by the proportion of actual foreign direct investment in GDP, the unit of the US dollar is adjusted to RMB based on the annual average exchange rate
Degree of openness	OPEN	When using the two indicators of the proportion of total import and export trade in GDP, the unit of the US dollar is adjusted to RMB based on the annual average exchange rate.
Green total factor productivity	GML	The input indicators are capital, labor, energy and water resources, the output indicators are actual GDP as the desirable output, and the industrial wastewater and waste gas emission is undesirable output

**Table 2:** Summary of average GML growth rate by province and year in the YRD from 2003 to 2019.

Year	Average Growth Rate of GML in Jiangsu Province	Average Growth Rate of GML in Zhejiang Province	Average Growth Rate of GML in Shanghai	Average Growth Rate of GML in Anhui Province	Mean Value of GML Growth Rate in YRD
2003–2004	0.972451	0.941206	1	0.902728	0.937531
2004–2005	0.979407	0.928238	1	0.977036	0.965256
2005–2006	0.932775	1.015935	0.9496	0.986268	0.976371
2006–2007	1.001462	1.012674	1.0429	0.976696	0.995816
2007–2008	1.066429	1.01081	1.0147	1.093313	1.060736
2008–2009	1.010115	0.970909	1.003	0.991999	0.992353
2009–2010	1.044536	1.069245	0.99219	1.039806	1.048043
2010–2011	1.020308	0.966238	1.7954	1.022486	1.025556
2011–2012	1.041635	0.983672	0.54311	0.989197	0.993461
2012–2013	1.037688	1.124828	1.0256	0.954114	1.028158
2013–2014	0.939304	0.850866	0.85125	1.011184	0.94148
2014–2015	0.988408	0.987608	0.94781	0.951701	0.972878
2015–2016	0.974235	1.119512	1.0664	1.078142	1.056009
2016–2017	0.910635	0.841714	1.1622	0.914653	0.899848
2017–2018	1.062916	1.11325	1.0013	1.019848	1.05811
2018–2019	1.080948	1.012893	0.99874	1.129626	1.079681
2013–2019	1.003953	0.99685	1.024638	1.002425	1.001955

**Table 3:** Summary of average BPC growth rate by province and year in the YRD from 2003 to 2019.

Year	Average Growth Rate of BPC in Jiangsu Province	Average Growth Rate of BPC in Zhejiang Province	Average Growth Rate of BPC in Shanghai	Average Growth Rate of BPC in Anhui Province	Mean Value of BPC Growth Rate in YRD
2003–2004	0.94516	0.953854	1	0.895824	0.929577
2004–2005	0.981932	0.961922	1	0.943616	0.962051
2005–2006	0.953116	0.993189	0.9496	0.993926	0.979708
2006–2007	1.002792	1.013592	1.0429	0.982313	0.998676
2007–2008	1.066208	1.014137	1.0147	1.091544	1.060869
2008–2009	0.987998	1.020192	1.003	1.00557	1.003859
2009–2010	1.034151	1.073091	0.99219	1.060014	1.053668
2010–2011	1.01863	0.936985	1.7954	1.002838	1.009508
2011–2012	1.024138	0.972136	0.54311	0.97269	0.978377
2012–2013	1.030305	1.137539	1.0256	0.960765	1.031823
2013–2014	0.969436	0.835637	0.85125	1.083184	0.975046
2014–2015	0.947829	0.989918	0.94781	0.906176	0.942866
2015–2016	0.995758	1.192436	1.0664	1.085362	1.085216
2016–2017	0.966127	0.928871	1.1622	0.948786	0.954146
2017–2018	1.074291	1.176891	1.0013	1.031944	1.083512
2018–2019	1.079854	1.027301	0.99874	1.077131	1.062713
2013–2019	1.004858	1.014231	1.024638	1.002605	1.006976

**Table 4:** Summary of average EC growth rate by province and year in the YRD from 2003 to 2019.

Year	Average Growth Rate of EC in Jiangsu Province	Average Growth Rate of EC in Zhejiang Province	Average Growth Rate of EC in Shanghai	Average Growth Rate of EC in Anhui Province	Mean Value of EC Growth Rate in YRD
2003–2004	1.030603	0.985326	1	1.007464	1.00868
2004–2005	0.99527	0.973789	1	1.039931	1.007051
2005–2006	0.978372	1.033568	1	0.992861	0.999362
2006–2007	0.999838	0.99912	1	0.994173	0.997439
2007–2008	0.999252	0.997039	1	1.00167	0.99962
2008–2009	1.023064	0.961415	1	0.988283	0.992388
2009–2010	1.010568	0.997486	1	0.986408	0.997372
2010–2011	1.001833	1.037915	1	1.023394	1.019883
2011–2012	1.017862	1.014	1	1.017733	1.01634
2012–2013	1.007531	0.983457	1	0.993271	0.995323
2013–2014	0.965725	1.019713	1	0.946419	0.973511
2014–2015	1.044835	0.997745	1	1.055799	1.035386
2015–2016	0.982194	0.953412	1	0.996111	0.980337
2016–2017	0.94732	0.913129	1	0.981236	0.952667
2017–2018	0.995302	0.951143	1	0.99121	0.981972
2018–2019	1.002077	0.987901	1	1.052734	1.017991
2013–2019	1.000103	0.987885	1	1.004294	0.998458

**Table 5:** Overall estimation results.

Variable	Equation (5)	Equation (6)	Equation (7)	Equation (8)
GML(−1)	−0.276264 *** (0.0000)	−0.278267 *** (0.0000)	−0.281241 *** (0.0000)	−0.288975 *** (0.0000)
ER	0.312018 *** (0.0000)	0.681847 *** (0.0000)	0.304650 *** (0.0000)	0.038850 (0.3053)
ER◊ER		−0.553697 *** (0.0000)		
FD			0.226589 *** (0.0000)	0.074708 (0.3050)
ER◊FD				2.825434 *** (0.0000)
AR(1)	0.0010	0.0009	0.0015	0.0008
AR(2)	0.4860	0.4679	0.6062	0.4709
Hansen-J	0.398402	0.347079	0.381883	0.374182

Note: *** is significant levels of 1% respectively, and the adjoint probability *p* is in brackets.

**Table 6:** Overall estimation results with control variables.

Variable	Equation (9)	Equation (10)	Equation (11)	Equation (12)
GML(−1)	−0.282957 *** (0.0000)	−0.275847 *** (0.0000)	−0.293918 *** (0.0000)	−0.295396 *** (0.0000)
ER	0.213317 *** (0.0000)	0.795267 *** (0.0000)	0.190924 *** (0.0000)	0.096351 (0.2681)
ER◊ER		−0.789845 *** (0.0000)		
FD			0.229440 ** (0.0215)	0.192864 * (0.0613)
ER◊FD				1.190128 ** (0.0376)
FI	0.071431 * (0.0813)	−0.239431 *** (0.0000)	−0.007582 (0.8779)	−0.035768 (0.6311)
FDI	−2.642130 *** (0.0001)		−2.409499 *** (0.0008)	−2.099880 *** (0.0044)
OPEN	0.034550 *** (0.0025)	0.034888 *** (0.0001)	0.037254 *** (0.0028)	0.036452 *** (0.0096)
AR(1)	0.0003	0.0000	0.0001	0.0000
AR(2)	0.0597	0.0986	0.0988	0.0683
Hansen-J	0.336793	0.31179	0.290363	0.270560

Note: *, ** and *** are significant levels of 10%, 5% and 1% respectively, and the adjoint probability *p* is in brackets.

## Data Availability

All the data are from the official websites of China Statistics Bureau, Shanghai Statistics Bureau, Jiangsu Statistics Bureau, Zhejiang Statistics Bureau and Anhui Statistics Bureau. http://www.stats.gov.cn/tjsj/ndsj/; https://tjj.sh.gov.cn/sjfb/index.html; http://tj.jiangsu.gov.cn/col/col83749/index.html; http://tjj.zj.gov.cn/col/col1525563/index.html; http://tjj.ah.gov.cn/ssah/qwfbjd/tjnj/index.html. (accessed on 24 November 2021). http://www.stats.gov.cn/; https://tjj.sh.gov.cn/; http://tj.jiangsu.gov.cn/; http://tjj.zj.gov.cn/; http://tjj.ah.gov.cn/ (accessed on 24 November 2021).
